# Au nanoparticle@hollow mesoporous carbon with FeCo/graphitic shell nanoparticls as a magnetically recyclable yolk–shell nanocatalyst for catalytic reduction of nitroaromatics

**DOI:** 10.1038/s41598-018-25795-w

**Published:** 2018-05-10

**Authors:** Yonghoon Hong, In Ae Choi, Won Seok Seo

**Affiliations:** Department of Chemistry, Sogang University, Seoul, 04107 Republic of Korea

## Abstract

We have developed a highly stable and magnetically recyclable yolk–shell nanocatalyst for catalytic reduction of nitroaromatics. This nanocatalyst is composed of a ~13 nm Au nanoparticle encapsulated in a hollow mesoporous carbon (hmC) shell with a diameter of ~120 nm and a thickness of ~15 nm. The hmC shell contains ~6 nm FeCo/graphitic carbon shell (FeCo/GC) nanoparticles. We have synthesized the Au@hmC-FeCo/GC nanocatalyst by thermal decomposition of Fe and Co precursors in silica of a solid core/mesoporous shell structure containing a Au nanoparticle within the core, subsequent ethylene chemical vapor deposition (CVD), and then removal of the silica by treatment with aqueous HF. The Au@hmC-FeCo/GC has superparamagnetism and high saturation magnetization (29.2 emu g^−1^) at room temperature. It also shows a type IV sorption isotherm, typical for mesoporous carbon (pore diameter = 3.5 nm), thereby ensuring ready accessibility to the Au core by substrates. We have shown that the Au@hmC-FeCo/GC catalyses the reduction of 4-nitrophenol and 4-nitrotoluene more efficiently than Au nanoparticles do, can be separated very quickly from the reaction mixture using an magnet, and can be reused for the same reduction reaction at least five times without loss of the initial level of catalytic activity.

## Introduction

Supported nanocatalysts, made by embedding metal nanoparticles (NPs) such as Au, Ag, Pd, and Pt on mesoporous supports, have attracted considerable attention for their high catalytic activities and efficiency toward various types of reactions (e.g., hydrogenation, oxidation-reduction, reforming, and coupling^1–3^), because they reduce aggregation between metal NPs and enable isolation and recovery of the very small metal NPs through filtration or centrifugation methods^4^. Mesoporous carbon, or silica with various structures, has been investigated as materials for catalyst support^5–7^. However, the inconvenience and inefficiency of the tedious filtration or centrifugation for recovering nanocatalysts have greatly limited practical applications of the supported nanocatalysts^8^. This drawback has been eliminated by development of magnetically recoverable supported nanocatalysts that can be simply and efficiently separated from reaction mixtures using an external magnetic field^4,9^. Accordingly, magnetic NPs coated with mesoporous silica or carbon, and mesoporous materials embedded with magnetic NPs have emerged as ideal catalyst supports^10^.

Among various magnetic materials, iron oxides have been most widely used as supports in catalysis because of their low cost and easy preparation^11^. However, iron oxides are not desirable for certain applications because they are inherently unstable in acidic media^12,13^, not inert in some catalytic processes^14–16^, not very strong in magnetic properties (*M*_s_ of bulk magnetite ≤92 emu g^−1^)^17^, and can provide an oxygen source in catalytic reactions, eventually giving unexpected product mixtures^18^. To circumvent such problems, graphitic carbon-coated metal NPs with very strong magnetic properties have been used as magnetic materials for magnetically recoverable catalysis. Reiser *et al*. produced magnetically recyclable catalysts for various reactions by immobilizing highly active palladium complexes on the surface of carbon-coated cobalt NPs^19,20^. Recently, our group also developed highly stable and magnetically recoverable mesoporous silica spheres embedded with FeCo/graphitic carbon shell (FeCo/GC) NPs (*M*_s_ of bulk FeCo = 235 emu g^−1^) for supporting phosphomolybdic acid as an acid catalyst and Pt NPs^12,21^. Despite the progress in magnetically recoverable catalysis, it is still highly desirable to improve the recyclability of the nanocatalyst system containing valuable metal NPs by developing highly stable magnetic catalyst supports.

Recently, yolk–shell or rattle-type nanostructures with a movable core inside a hollow shell have generated much interest in a variety of applications including catalysis, energy storage and conversion, and drug delivery. This intense interest is due to their unique features, such as high specific surface area, large void space, low density, and multi-functionality^22–27^. The hollow mesoporous shell of these structures can prevent the aggregation of neighboring cores and effectively protect the core from escaping to the outside while allowing the fast diffusion of reactants and products. Moreover, it provides a void space in which catalytic reactions can occur. Therefore, many yolk–shell nanomaterials with a catalytic metal NP encapsulated in a hollow mesoporous sphere, such as Au (or Pt) NP@hollow mesoporous carbon and Au NP@hollow mesoporous silica, have been synthesized and successfully applied for heterogeneous catalysis^28–32^. It would be very useful and practical to introduce superparamagnetic NPs into the yolk–shell nanocatalyst system for convenient magnetic recovery. However, until now, only a few yolk–shell nanocatalysts containing magnetic NPs have been developed. The method most frequently used to prepare such nanocatalysts has been the formation of magnetic core–hollow porous shell structures such as Fe_3_O_4_@hollow polymer (or carbon)^33^, Fe_3_O_4_@hierarchical nickel silicate^34^, and SiO_2_@Fe_3_O_4_/carbon double-layered shell^35^; with subsequent loading of catalytic metal NPs within the mesoporous shell and the interior cavity through reduction of metal salt precursors. However, the catalysts obtained by this method have catalytic metal NPs that are not fully encapsulated within the hollow shells and thus may drop away from the shells. Yao *et al*. prepared yolk–shell composites with a movable iron oxide core and mesoporous silica shell, together with Pd NPs anchored on the inner silica surface^13^, for the catalytic reduction of 4-nitrophenol. However, these composites did not have a single catalytic NP inside the hollow mesoporous sphere, but instead, hosted some catalytic NPs in the void space of the yolk–shell structure. In this case, the catalytic NPs could not be completely prevented from aggregating. Very recently, Lin and Doong fabricated Au@Fe_3_O_4_ yolk–shell nanocatalysts for the catalytic reduction of nitroarenes^36^. However, the magnetic yolk–shell nanocatalysts were not magnetically recyclable because of their small size, water-solubility, and low magnetic moment. Therefore, it is still necessary to develop a facile strategy for fabricating yolk–shell nanomaterials composed of a single catalytic core and a magnetic hollow mesoporous shell with excellent catalytic efficiency, long-term stability, and suitability for magnetic recycling.

**Figure 1:**
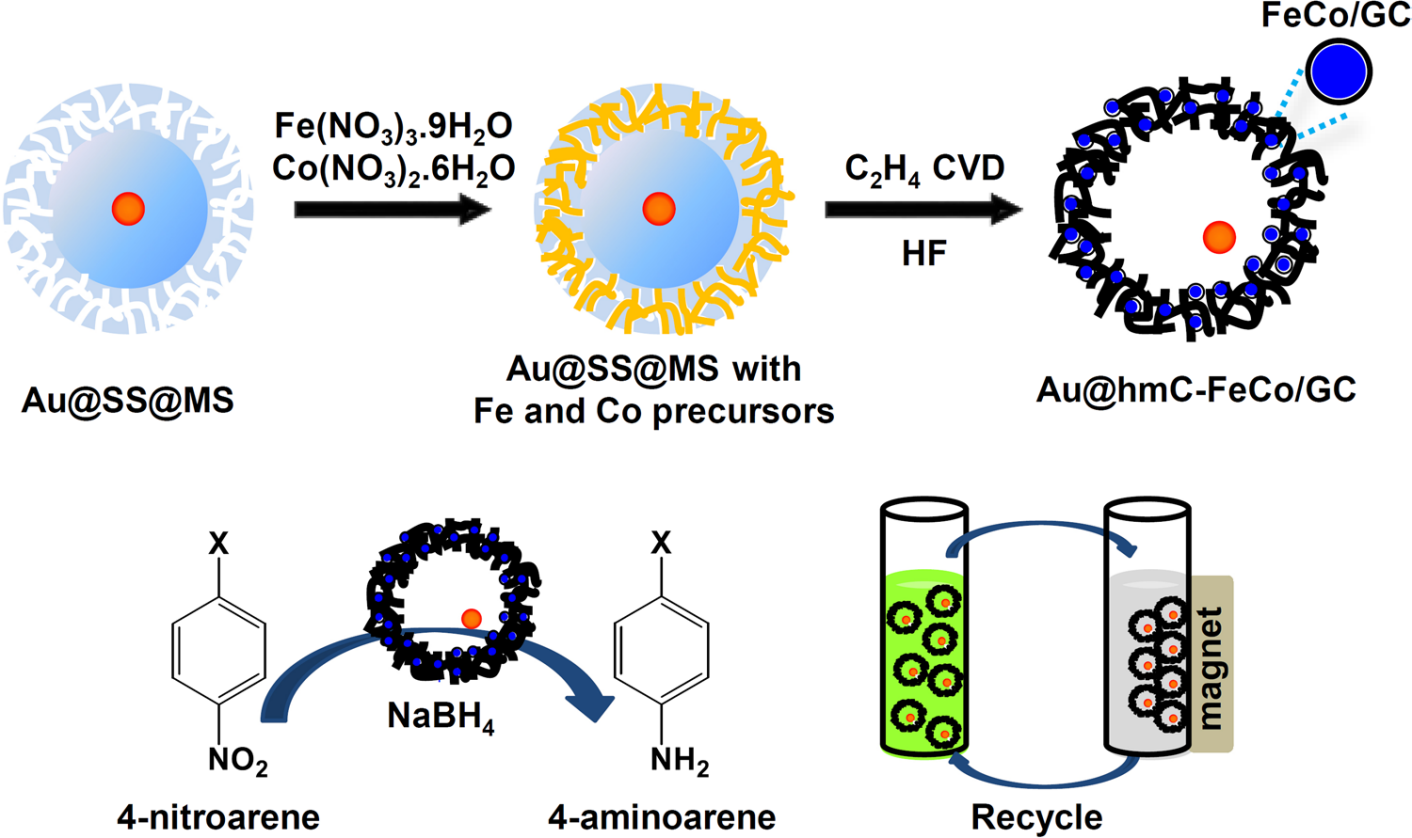
Schematic illustration for the preparation of Au@hmC-FeCo/GC, catalytic reduction of 4-nitroarene to 4-aminoarene by the Au@hmC-FeCo/GC, and separation of the Au@hmC-FeCo/GCs by using a magnet.

Herein, we report a process for facile synthesis of a highly stable and magnetically recyclable yolk–shell nanocatalyst. It is composed of a Au NP encapsulated in a hollow mesoporous carbon (hmC) shell containing FeCo/graphitic carbon shell (FeCo/GC) NPs (thus, Au@hmC-FeCo/GC). The schematic strategy for the preparation of Au@hmC-FeCo/GC is illustrated in Fig. 1. The obtained Au@hmC-FeCo/GC possesses superparamagnetism, very high saturation magnetization (29.2 emu g^−1^) at room temperature, and uniformly accessible meso-channels (3.5 nm) that allow rapid diffusion of small molecules. The hmC embedded with FeCo/GC NPs stabilizes the Au core by preventing the coalescence of the Au NPs, and provides a void space for catalytic reactions. Figure 1 illustrates the catalytic reduction of 4-nitroarene that occurs on the surface of the Au core in the presence of NaBH_4_, and the convenient and efficient recovery of the Au@hmC-FeCo/GC using a magnet. We have shown that Au@hmC-FeCo/GC works as an excellent recyclable nanocatalyst system that catalyses the reduction of nitroaromatics. This is the first demonstration of such highly stable and efficiently recyclable yolk–shell nanocatalysts with a single catalytic core encapsulated in a magnetic hollow mesoporous shell.

## Results and Discussion

As illustrated in Fig. 1, we used a solid silica core/mesoporous silica shell (SS@MS) nanosphere containing a Au-NP inside the core (Au@SS@MS) as a template for the growth of hmC and FeCo/GC NPs. The 12.4 ± 1.5 nm Au NPs and Au@SS@MSs with silica core diameter of ~100 nm and shell thickness of ~20 nm were synthesized by a slight modification of a previously reported method^37^. As confirmed in TEM images of the Au NPs (see Supplementary Fig. S1a) and Au@SS@MSs (Supplementary Fig. S1b), the Au NPs retained the size and size distribution of the original Au seeds after being covered with silica layers. Most (90%) of the Au@SS@MS nanospheres contained a single Au NP. The X-ray diffraction (XRD) spectra of Au NPs and Au@SS@MSs in Supplementary Fig. S1c confirm the face-centered-cubic (fcc) crystal structure of the Au.

The common procedure used to synthesize hmC is based on the growth of carbon inside the mesoporous silica shell and subsequent removal of the siliceous components by treatment with aqueous HF. In previous reports, the hmC shells were produced by the carbonization of loaded polymers such as phenol resin^27,37^, polyacrylonitrile^38,39^, polydopaminine^40^, and biomass^41^. However, carbon materials fabricated by these carbonization methods were generally amorphous or weakly graphitized. This required high temperatures and long reaction times for the formation of high-quality carbon with graphitic crystallinity and good structural strength^42,43^. Moreover, carbon shells tend to have porous structures because they are formed from the thermal decomposition of the given carbon sources without being supplied with additional precursors, leading to a weak structure that is easily broken^40,44^. On the other hand, carbon materials produced by the chemical vapor deposition (CVD) of carbon precursors such as methane, ethylene, benzene, and alcohols have characteristics of imperviousness, high purity and hardness^45,46^. Also, their structural features (e.g., density or porosity) can easily be controlled by the reaction time and type of carbon sources^42,43^. In this work, we prepared hmC shells with a Au NP in each shell (Au@hmCs) using the CVD method and ethylene as the carbon source. This was done in the presence of a silica template at the reaction temperature of 800 °C, and with the ethylene flow time of 20 min. We have found that the prepared carbon shells are thin and they include partially broken ones when the ethylene flow time is decreased to 15 min (Supplementary Fig. S2a). In contrast, the carbon shells grown using the ethylene flow time of 25 min are thick and the products include some carbon materials formed outside the silica templates (Supplementary Fig. S2b). Au@hmCs containing FeCo/GC NPs inside the hmC shells (Au@hmC-FeCo/GCs) were obtained through the same ethylene CVD process, but with the silica templates containing Fe and Co precursors, Fe(NO_3_)_3_·9H_2_O and Co(NO_3_)_2_·6H_2_O. We loaded the metal precursors (1.2 mmol with a Fe:Co molar ratio of 58:42) into the silica template (1.0 g) by impregnation in a methanol solution and evaporation of methanol. FeCo alloy NPs were formed by the thermal decomposition of the metal precursors during the heating process under a reducing atmosphere created by the H_2_ flow. Further ethylene CVD at 800 °C promoted deposition of graphitic carbon layers over the FeCo NPs grown inside the mesoporous silica shell and deposition of carbon materials inside the mesopores.

**Figure 2:**
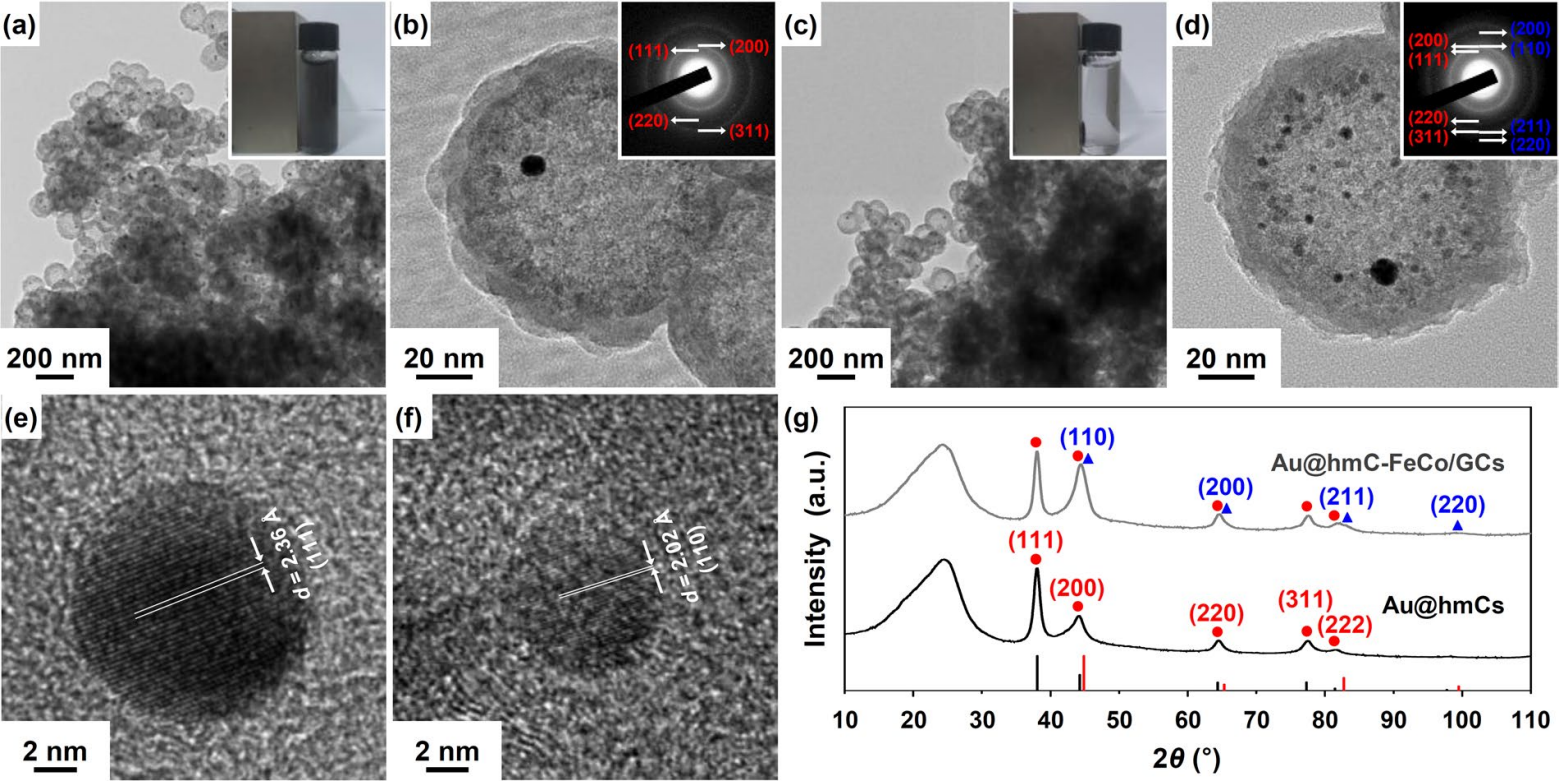
TEM images of (**a**,**b**) Au@hmCs and (**c**,**d**) Au@hmC-FeCo/GCs. Insets in (**a**,**c**) are photographs of Au@hmCs (**a**) and Au@hmC-FeCo/GCs (**c**) in water after the placement next to a NbFeB magnet for 30 sec. Insets in (**b**,**d**) are SAED patterns. High resolution TEM images of the (**e**) Au and (**f**) FeCo/GC NPs in Au@hmC-FeCo/GC. (**g**) XRD patterns of Au@hmCs and Au@hmC-FeCo/GCs (red circles and blue triangles indicate the reflections of fcc-Au and bcc-FeCo, respectively).

Figure 2a–d, show the transmission electron microscopy (TEM) images of Au@hmCs and Au@hmC-FeCo/GCs, respectively. The magnified images in Fig. 2b,d clearly show the ~13 nm Au NPs (13.2 ± 2.2 nm for Au@hmCs and 13.3 ± 1.8 nm for Au@hmC-FeCo/GCs) encapsulated in hmC shells with a diameter of ~120 nm and a thickness of ~15 nm. The small dots that originated from the 6.2 ± 1.5 nm FeCo/GC NPs are clearly observed in Fig. 2d. The selected-area electron diffraction (SAED) pattern of the Au@hmCs in the inset of Fig. 2b is consistent with fcc-Au with reflections due to the (111), (200), (220), and (311) planes. The SAED pattern of the Au@hmC-FeCo/GCs in the inset of Fig. 2d includes reflections from body-centered-cubic (bcc) FeCo due to the (110), (200), (211), and (220) planes, in addition to those of fcc-Au. The high-resolution TEM images of Au and FeCo/GC NPs in Fig. 2e,f clearly show the lattice fringes of the fcc-Au (*d* spacing = 2.36 Å for a (111) reflection) and the bcc-FeCo (*d* spacing = 2.02 Å for a (110) reflection), respectively. The crystal structures of bcc-FeCo and fcc-Au were also confirmed by powder X-ray diffraction (XRD, Fig. 2g). The crystallite sizes of the Au NPs determined for the (111) reflections of the XRD data using the Debye–Scherrer equation, were both 13.2 nm, which well matched the mean diameter estimated from the TEM images. This implies the single-crystalline nature of each Au-NP. The increase (~0.8 nm) of the Au size relative to that in Au@SiO_2_-mSiO_2_ is probably due to the coalescence of the two Au NPs originally attached to each other in the silica templates.

**Figure 3:**
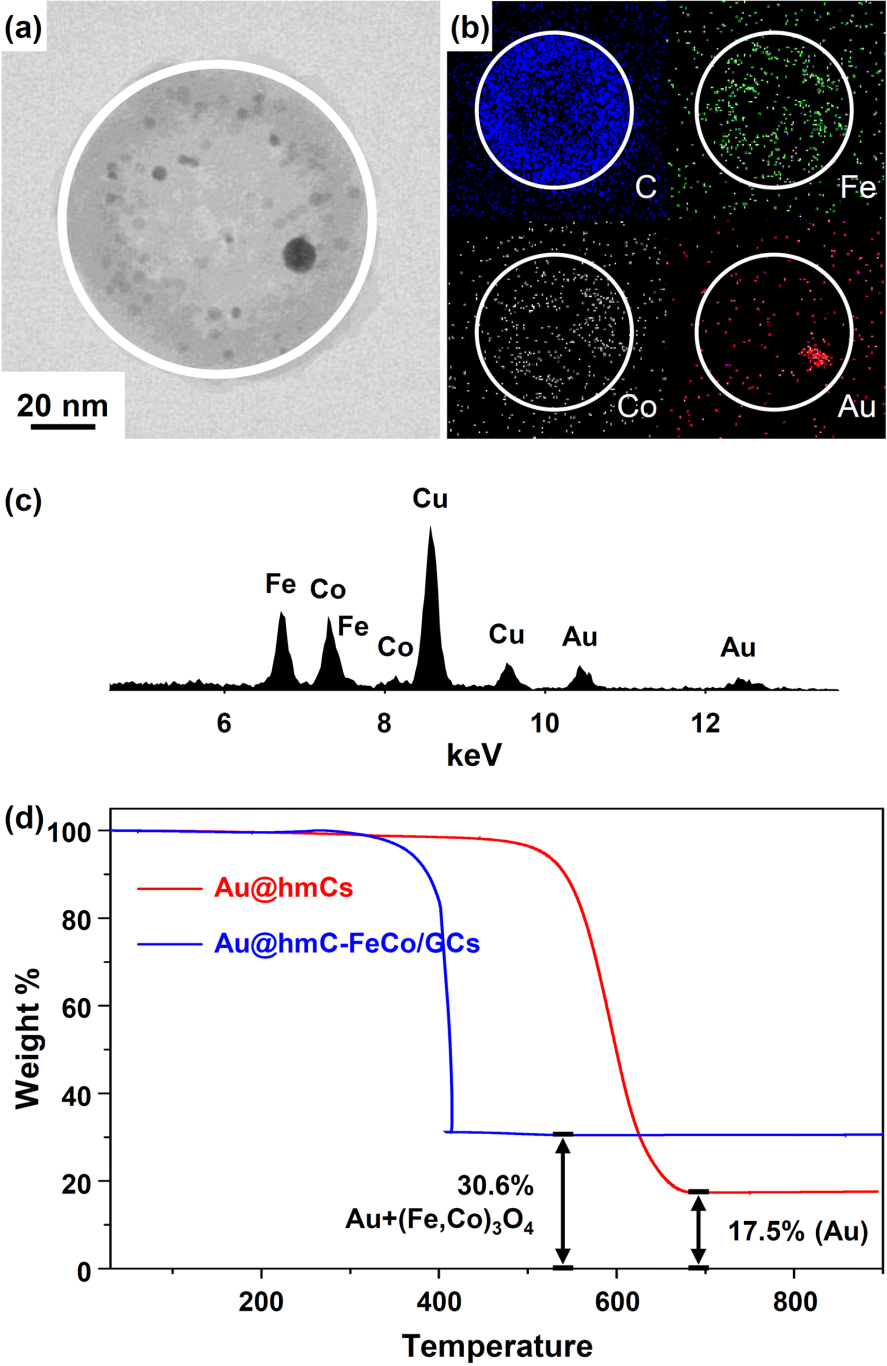
(**a**) STEM and (**b**) STEM-EDX elemental mapping (blue = carbon, green = Fe, white = Co, and red = Au) images, and (**c**) EDX spectrum of Au@hmC-FeCo/GCs. Copper is from the TEM grids. (**d**) TGA profiles for Au@hmCs and Au@hmC-FeCo/GCs.

Elemental distribution maps (Fig. 3b) of C, Fe, Co, and Au were obtained from the same region of a Au@hmC-FeCo/GC (Fig. 3a) using a TEM equipped with an energy-dispersive X-ray (EDX) analyzer. As shown in Fig. 3b, the three elements, C, Fe, and Co are uniformly distributed in the sample, indicating that FeCo/GC NPs were well dispersed within hmC shells without any agglomeration, while Au was placed at the NP location shown in the Scanning TEM (STEM) image in Fig. 3a. The weight Au:Fe:Co ratio of the Au@hmC-FeCo/GCs, obtained by analyzing the corresponding elemental peaks in the EDX spectrum (Fig. 3c), was 48.2:25.0:26.8. We performed TGA in air to determine the weight percent of the components (Au, Fe, Co, C) in Au@hmCs and Au@hmC-FeCo/GCs (Fig. 3d). It is well known that FeCo alloy is rapidly oxidized to spinel ferrite (Fe,Co)_3_O_4_ at 350 °C and above^47^. The Au/C weight percent of Au@hmCs and the Au + (Fe,Co)_3_O_4_/C weight percent of Au@hmC-FeCo/GCs measured by TGA were 17.5/82.5 and 30.6/69.4 wt%, respectively. The Au/Fe/Co/C weight percent of Au@hmC-FeCo/GCs, determined by using the weight Au:Fe:Co ratio and the Au + (Fe,Co)_3_O_4_/C weight percent obtained from EDX and TGA^48,49^, respectively, was 13.0/6.8/7.2/73.0 wt% (Table 1). We confirmed that the weight percentages of Fe and Co were very close to those (Fe: 6.6%, Co: 7.4%) obtained using a calcination/HCl/UV-vis method reported previously^50^.

**Table 1:** EDX data for Au@hmC-FeCo/GCs, TGA data for Au@hmCs and Au@hmC-FeCo/GCs, and Fe and Co wt. percentages obtained by using a calcination/HCl/UV-vis method for Au@hmC-FeCo/GCs

Samples	Au@hmCs	Au@hmC-FeCo/GCs
EDX	**—**	Weight Au:Fe:Co ratio 48.2:25.0:26.8
TGA	Au/C wt% 17.5:82.5	Au + (Fe,Co)_3_O_4_/C wt% 30.6/69.4
Estimated	**—**	Au/Fe/Co/C wt% 13.0/6.8/7.2/73.0
Calcination/HCl/UV-vis	**—**	Fe/Co wt% 6.6/7.4

.

**Figure 4:**
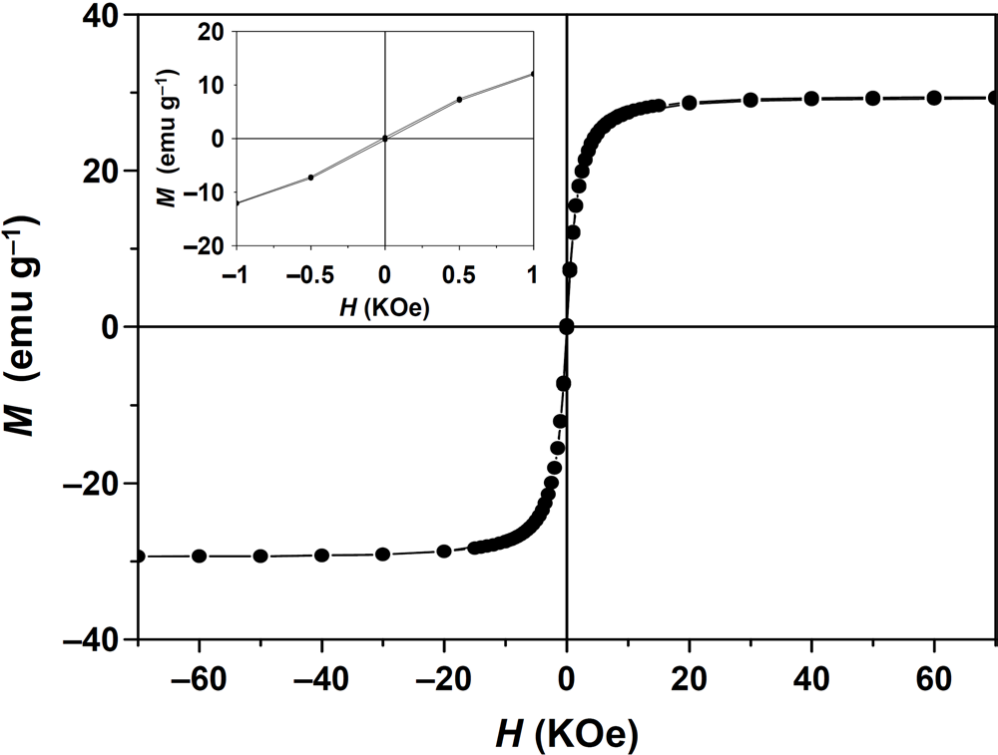
Field-dependent magnetization curves of Au@hmC-FeCo/GCs measured at 300 K. The inset shows the loop on an enlarged x-axis scale.

As shown in Fig. 4, field-dependent magnetic measurements were carried out with a superconducting quantum interference device-vibrating sample magnetometer (SQUID-VSM). The Au@hmC-FeCo/GCs did not display any coercivity at 300 K (Fig. 4, inset), which is indicative of superparamagnetic characteristics. It is very important to maintain the superparamagnetic property of magnetic nanocatalysts for applications because it prevents their magnetic aggregation and facilitates redispersion upon removal of an external magnetic field. The Au@hmC-FeCo/GCs had a very high saturation magnetization of 29.2 emu g^−1^, which mostly originated from the FeCo/GC NPs when the weight percentages (total 14.0 wt%) of Fe and Co in the sample were considered. The saturation magnetization is equal to the value of samples with 32 wt% bulk iron oxides. As shown in the inset of Fig. 2c, the Au@hmC-FeCo/GCs in water in a 4 mL vial were almost completely collected by a NbFeB magnet within 30 sec due to the high saturation magnetization, thus resulting in a clear solution, in contrast to the case of Au@hmCs in the inset of Fig. 2a.

FeCo/GC NPs not only exhibit 2–3 times higher saturation magnetization values than do iron oxide NPs^51^, but they also exhibit superior chemical stability against acid etching thanks to the graphitic carbon shells encapsulating the FeCo cores. This is in contrast to magnetic metals or metal oxides such as Fe, Co, iron oxides, and ferrites^50^. Actually, the Au@hmC-FeCo/GCs were very stable in a 35% HCl solution over a monitoring period of six months (Supplementary Fig. S3a). Moreover, the Au@hmC-FeCo/GCs were still stable even under severe conditions of heating at 300 °C in air for 1 h (Supplementary Fig. S3b). Only after being heated at 550 °C in air for 1 h, did the Au@hmC-FeCo/GCs turn the solution green right after addition of the HCl solution owing to the etching of Fe and Co (Supplementary Fig. S3c).

**Figure 5:**
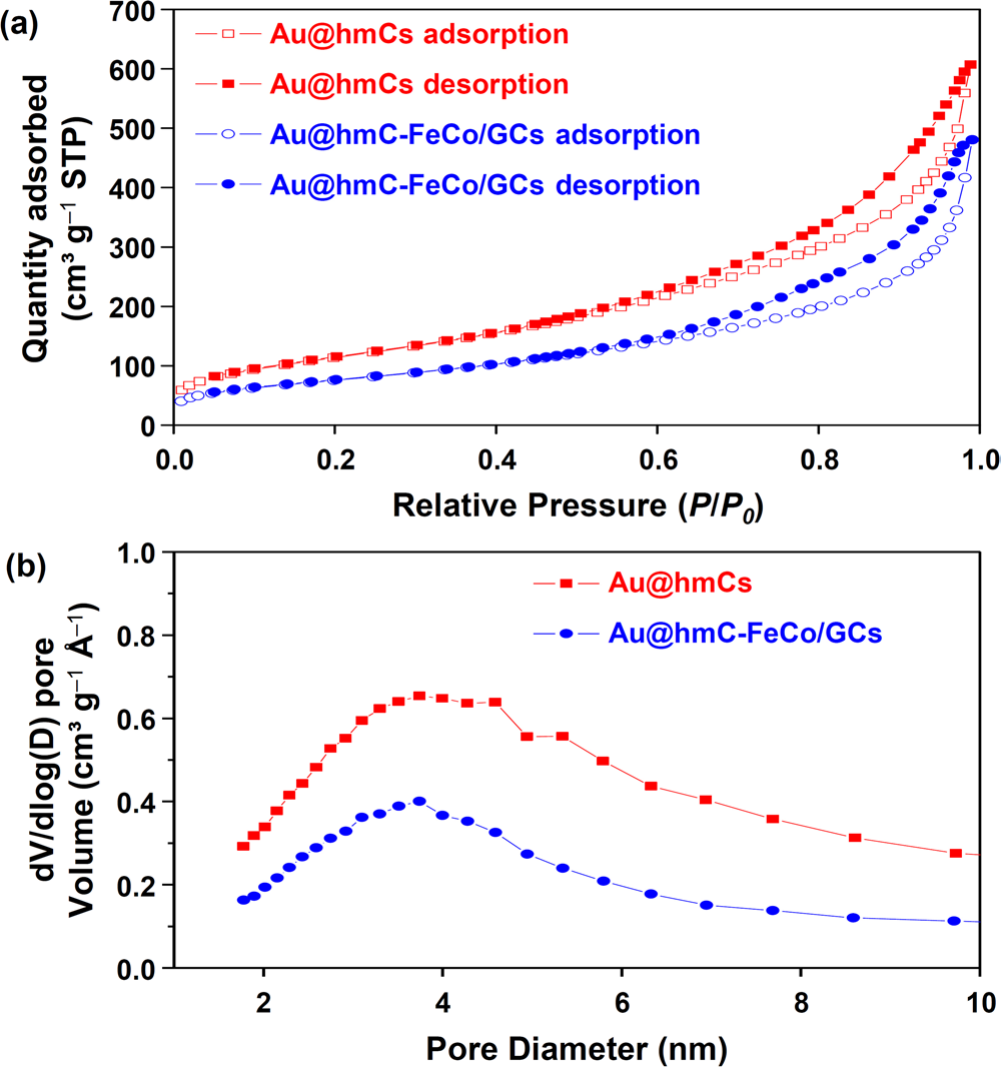
(**a**) Nitrogen sorption isotherms and (**b**) Pore size distributions of Au@hmCs and Au@hmC-FeCo/GCs.

The surface area and porosity of the Au@hmCs and Au@hmC-FeCo/GCs were investigated using N_2_ adsorption–desorption isotherms (Fig. 5). Supplementary Table S1 summarizes the physisorption data. Both samples show a type IV adsorption isotherm with a H_2_-type hysteresis loop, which is characteristic of mesoporous carbon synthesized using mesoporous silica templates^37^. The BET (Brunauer–Emmett–Teller) surface area, total pore volume, and the BJH (Barrett–Soyner–Halenda) average pore diameter for the Au@hmC-FeCo/GCs were 276 m^2^ g^−1^, 0.74 cm^3^ g^−1^, and 3.5 nm, respectively, which are smaller than those of Au@hmCs (419 m^2^ g^−1^, 0.93 cm^3^ g^−1^, and 3.7 nm). This is mainly ascribed to the increase in the density of materials caused by the loading of FeCo/GC NPs or the catalytic function of FeCo NPs. The FeCo NPs, formed *in situ* by the thermal decomposition of the metal precursors, could promote the formation and graphitization of carbon products in the CVD process, leading to the synthesis of carbon materials with high density and rigidity. Because the pores of the hmCs are mainly formed by the removal of mesoporous silica templates, they are larger than the mesopores of the silica templates. Moreover, the decrease in the pore diameter of hmCs by the loading of FeCo/GC NPs was only 0.2 nm. Therefore, we expect that the Au@hmCs and Au@hmC-FeCo/GCs possess porosity enough for the fast diffusion of reactants and products during catalytic reactions.

**Figure 6:**
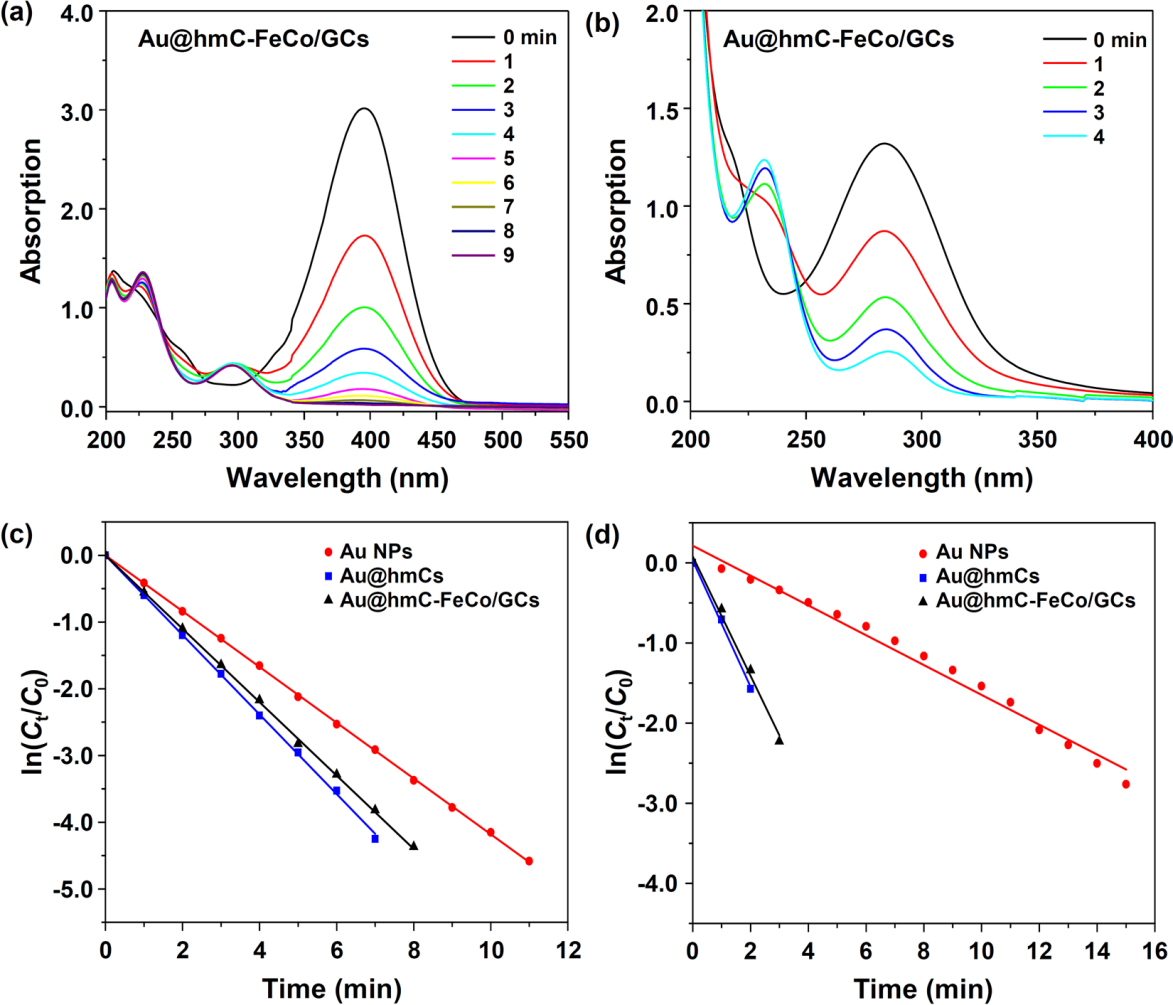
(**a**,**b**) Time-dependent UV-vis spectral changes of the reaction mixture catalysed by Au@hmC-FeCo/GCs for 4-nitrophenol (**a**) and 4-nitrotoluene (**b**). (**c**,**d**) Plot of ln (*C*_0_/*C*_t_) versus reaction time for each nanocatalyst for the reduction reactions of 4-nitrophenol (**c**) and 4-nitrotoluene (**d**).

We chose the Au-catalysed reduction of 4-nitrophenol to 4-aminophenol in the presence of NaBH_4_ as a model reaction to demonstrate the use of our Au@hmC-FeCo/GCs as a magnetically recoverable nanocatalsyt and to compare their catalytic properties with those of Au@hmCs and Au NPs. The reduction reaction does not occur without the Au@hmC-FeCo/GC nanocatalysts, as evidenced by the constant absorption peak of 4-nitrophenol at 400 nm. However, when the nanocatalysts were introduced into the solution, the absorption at 400 nm quickly decreased and the absorption of 4-aminophenol at 295 nm increased concomitantly (Fig. 6a). The reduction of 4-nitrophenol to 4-aminophenol was completely finished within ~10 min. To examine any catalytic effects of hmC and FeCo/GC NPs on the reduction reaction, we prepared hmC-FeCo/GCs (Supplementary Fig. S4a,b) using a SS@MS nanosphere without a Au NP inside the core as a template and performed the reduction reaction with the hmC-FeCo/GCs under the same conditions. It has been reported that Co NPs show some catalytic properties for the 4-nitrophenol reduction reaction with NaBH_4_^52^. However, in this case, the hmC-FeCo/GCs showed no catalytic activity (Supplementary Fig. S4c), which clearly demonstrates that all the FeCo NPs were fully coated by graphitic carbon shells and that the catalytic functions originated only from Au.

The ratio of *C*_t_/*C*_0_, where *C*_t_ and *C*_0_ are 4-nitrophenol concentrations at the specific time intervals and the initial time, respectively, was measured from the ratio of the respective absorbances, *A*_t_/*A*_0_. Figure 6c shows a linear correlation between ln(*C*_t_/*C*_0_) and the reaction time at room temperature, indicating that the reaction follows pseudo-first-order kinetics. The rate constant (*k*) at room temperature was calculated from the slope to be 0.55 min^−1^, which is among the highest values reported in the literature (Supplementary Table S2). Similar reduction reactions were carried out with Au NPs (Supplementary Fig. S5a) and Au@hmCs (Supplementary Fig. S5b) for comparison, and the rate constants obtained were 0.42 min^−1^ and 0.60 min^−1^, respectively. The catalytic properties of Au@hmCs and Au@hmC-FeCo/GCs are similar, although the reaction rate of Au@hmCs is slightly faster, probably due to their slightly larger pore size. However, the Au@hmCs and Au@hmC-FeCo/GCs showed significantly faster reaction rates than Au NPs, which can be explained by the characteristic of the yolk–shell structure of the nanocatalysts, such as the facile diffusion of 4-nitrophenol through the mesoporous carbon shell, the large free-reaction voids inside the carbon shell, and the highly stable Au NPs in each carbon shell^29,30^.

We further investigated the catalytic activities of the Au@hmC-FeCo/GCs, Au NPs, and Au@hmCs towards the reduction of 4-nitrotoluene (a nitroaromatic compound with a hydrophobic substituent) to examine the functionality of hmC. As shown by the UV-vis spectra in Fig. 6d and Supplementary Fig. S5c,d, and the pseudo-first-order kinetic plots in Fig. 6d, the reaction rates followed the same sequence, Au@hmC >Au@hmC-FeCo/GC > Au NP, as that for the reduction of 4-nitrophenol to 4-aminophenol. However, the reaction rates of the Au@hmCs and Au@hmC-FeCo/GCs (*k* = 0.79 min^−1^ and 0.74 min^−1^, respectively) were observed to be much faster than those of Au NPs (*k* = 0.19 min^−1^) compared with the case of 4-nitrophenol, which can be attributed to the hydrophobic nature of the hmC with large specific surface area. Previously, Xu *et al*. reported that super-hydrophobic yolk–shell catalysts with Au NPs showed high reaction rate towards the reduction of nitroaromatic compounds because of their high affinity towards organic substrates in aqueous phase^32^. The higher concentration of nitroaromatic compounds inside the hmC would enhance the reaction rate.

**Figure 7:**
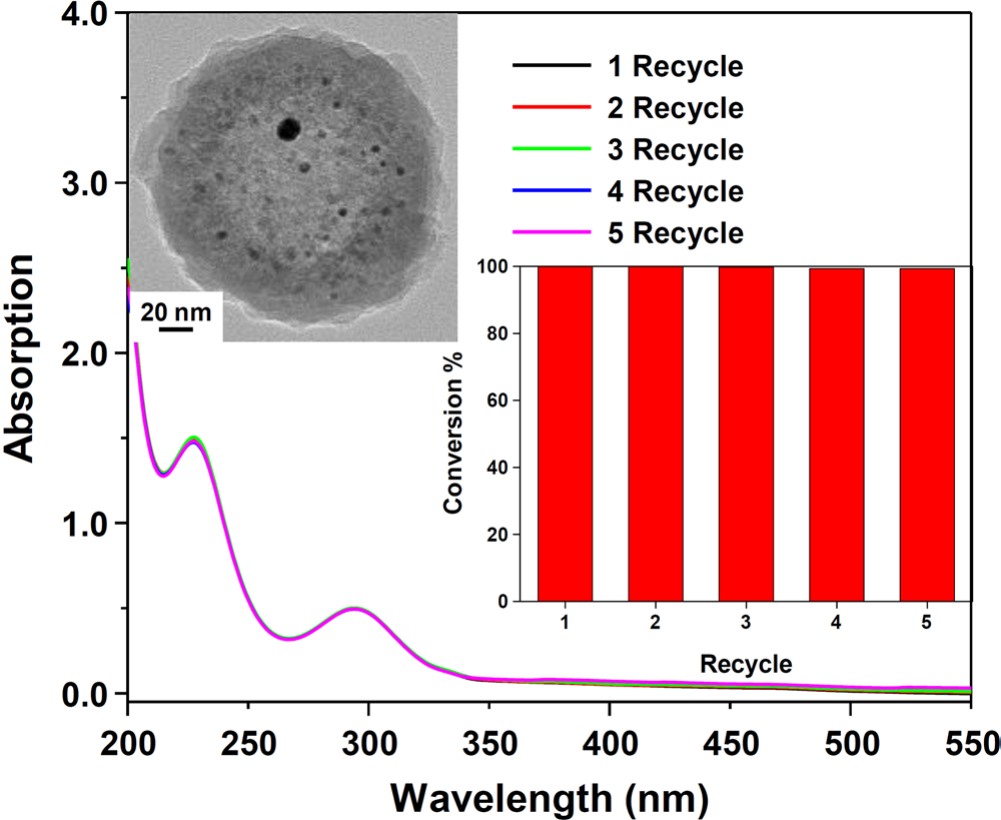
UV-vis spectra of 4-nitrophenol 10 min after the addition of recycled Au@hmC-FeCo/GCs for the five consecutive reduction reactions. Inset: a TEM image of the Au@hmC-FeCo/GC obtained after the fifth recycle experiment and a graph of the conversion of 4-nitrophenol in 10 min versus the number of catalyst recycles.

To demonstrate the stability of Au@hmC-FeCo/GCs, we performed the 4-nitrophenol reduction reaction under the same conditions with the recycled Au@hmC-FeCo/GCs, five times consecutively. In each cycle, 10 min after the addition of the recycled Au@hmC-FeCo/GCs, the reaction was stopped by almost completely separating the nanocatalysts from the solution within 30 s using a NdFeB magnet. Then, the nanocatalysts were rinsed with deionized (DI) water, and dispersed into DI water for the next cycle of catalysis. As shown by the UV-vis spectra (Fig. 7) and histograms (Fig. 7, inset), the Au@hmC-FeCo/GCs were successfully recycled and reused five more times, all with conversions of nearly 100%. A TEM image of the Au@hmC-FeCo/GC obtained after the fifth recycle experiment (Fig. 7, inset) revealed that there was no change in the structure of the nanocatalysts. This further confirmed the high stability of the Au@hmC-FeCo/GC nanocatalysts. On the other hand, Au NPs were found to be severely aggregated after the first reduction reaction (Supplementary Fig. S6), demonstrating the low stability of Au NPs. Obviously, the presence of hmC was sufficient to stabilize the Au NPs by preventing their aggregation. At the same time, the chemical inertness, good mechanical stability, and superior magnetic properties of hmC-FeCo/GCs render the Au@hmC-FeCo/GCs with high catalytic stability and convenience of practical use, giving the hmC-FeCo/GCs encapsulating a catalytic metal NP in each sphere, as presented herein, great potential as highly stable and magnetically recoverable nanocatalysts.

## Conclusions

In summary, we demonstrated the successful synthesis of a highly stable and magnetically recyclable yolk–shell nanocatalyst (Au@hmC-FeCo/GC) composed of a Au core NP and a hollow mesoporous carbon shell containing FeCo/GC NPs created by ethylene CVD using a Au core/silica shell nanosphere as the template. The Au@hmC-FeCo/GCs showed superparamagnetic properties and very high long-term stability in a 35% HCl solution. The Au@hmC-FeCo/GCs exhibited more efficient catalytic performance in the reduction of 4-nitrophenol and 4-nitrotoluene compared with the Au NPs, because of the yolk–shell structure characteristics and their hydrophobic nature. In addition, due to the very high saturation magnetization of 29.2 emu g^−1^, the nanocatalysts could be separated from the reaction solutions within 30 s using a magnet and they could be reused further for the same reduction reaction at least five times with no significant decrease in the initial level of catalytic activity. Our yolk–shell nanocomposite system may offer a useful platform for highly stable and magnetically recyclable nanocatalysts for various heterogeneous catalytic reactions. This synthesis method could be readily extended to prepare a number of yolk–shell structured magnetic nanocatalysts with catalytically important metal cores (i.e., Pt, Pd, and Rh).

## Methods

### Chemicals

Gold(III) chloride trihydrate (ACS reagent, ≥49.0% Au basis, Sigma-Aldrich), sodium citrate tribasic dihydrate (ACS reagent, ≥99.0%, Sigma-Aldrich), polyvinylpyrrolidone (PVP, powder, average M_w_ ~55,000, Aldrich), ethyl alcohol (99.9%, Jin Chemical Co., Ltd), ammonia water (28%, Jin Chemical Co., Ltd), tetraethyl orthosilicate (98%, Sigma-Aldrich), octadecyltrimethoxysilane (≥85.0%, TCI), iron(III) nitrate nonahydrate (99.99%, Aldrich), cobalt(II) nitrate hexahydrate (99.999%, Aldrich), sodium borohydride (99%, Aldrich), hydrochloric acid (35.0%, Jin Chemical Co., Ltd), hydrofluoric acid (48.0–51.0%, J.T.Baker), and all other reagents purchased from commercial sources were used as obtained without further purification.

### Synthesis of Au NPs

100 *μ*L of a HAuCl_4_ stock solution (1.00 g/5.00 mL of DI water) was added to DI water (100 mL). The resulting solution was stirred under reflux. Then, a sodium citrate solution (3.00 mL, 5 wt.%) was added and the system was stirred for 10 min. The resulting colloid was cooled to room temperature and 13 nm Au NPs were obtained. A fresh solution of PVP (0.33 mL, 0.013 g/1.00 mL) was added to a previously-prepared Au colloid. The resulting mixture was stirred for 24 h to allow complete adsorption of the polymer on the Au surface. Then, the solution was filter-centrifuged and the excess ligand was removed. The volume of the concentrated Au colloid was then adjusted to 12.0 mL using dilution with DI water.

### Synthesis of Au@SS@MSs

Au@SS@MSs were prepared through a modified stöber process^37^. The Au colloid was ultrasonicated for 10 min, followed by addition of ethanol (80 mL) and concentrated ammonia water (1.60 mL, 28 wt% NH_3_ in water). Afterwards, a solution of TEOS (0.60 mL) was added, and then the resulting colloid was stirred for additional 3 h at 0 °C. Continuously, the concentrated ammonia water (2.00 mL) and C_18_TMS (0.36 mL) in TEOS (0.90 mL) were added. After being stirred for additional 2 h at 0 °C, the resulting colloid was centrifuged, washed with DI water and ethanol, and dried at 80 °C. To remove all organic residues incorporated in the silica, we sintered the powder in air at 550 °C for 6 h.

### Synthesis of Au@hmCs

The as-prepared Au@SS@MSs (0.20 g) were used for ethylene CVD in a tube furnace. We heated the powder sample in a H_2_ flow to reach 800 °C for 11 min and then subjected it to an ethylene flow of 50 cm^3^ min^−1^ for 20 min. On cooling, we washed the product with a 2N HF (1:1 volume ratio of ethanol and DI water) to dissolve the silica template. Finally, we obtained the Au@hmCs by centrifugation and thoroughly washed them with ethanol and DI water.

### Synthesis of Au@hmC-FeCo/GCs

We impregnated Au@SS@MSs (1.00 g) with Fe(NO_3_)_3_∙9H_2_O (0.281 g, 0.696 mmol) and Co(NO_3_)_2_∙6H_2_O (0.147 g, 0.504 mmol) in methanol (50 mL) and sonicated them for 2 h. After removal of the methanol by evaporation and drying at 80 °C, we ground the resulting powder and typically used 0.20 g for ethylene CVD in a tube furnace. Au@hmC-FeCo/GCs were obtained through the same CVD procedure as Au@hmCs.

### Catalytic reduction of 4-nitroarenes

The rate constants of 4-nitroarene reduction reactions were evaluated using UV-vis spectroscopy. In a typical reaction, 4-nitroarene (0.08 mL, 0.01 M) was mixed with a Au nanocatalyst (0.05 mL, 7.92 × 10^−5^ M of Au) and DI water (3.95 mL) at room temperature in a glass vial. A fresh prepared aqueous solution of NaBH_4_ (0.80 mL, 0.10 M) was then added with constant stirring. Then, UV-vis adsorption spectra were recorded with time to monitor the change in the reaction mixture.

### Characterization

We characterized the synthesized materials by X-ray diffraction (XRD, Rigaku Miniflex II (4.5 kW) diffractometer using Cu-Kα radiation at 30 kV and 15 mA), transmission electron microscopy (TEM, JEOL JEM-2100F operated at 200 KV) with selected area electron diffraction (SAED) patterns, energy dispersive X-ray spectroscope (EDX), and scanning TEM-EDX (STEM-EDX). Samples for TEM and STEM investigation were prepared by dropping the diluted samples in ethanol on a 300 mesh carbon support copper grid (Ted Pella, Inc.). The magnetic properties were measured using a superconducting quantum interference device (SQUID, Quantum Design MPMS SQUID-VSM). The sample was collected in a gelatin capsule and inserted into the magnetometer. The hysteretic loops were obtained at 300 K in a magnetic field varying from +7 to −7 T. Thermogravimetric Analysis (TGA, TA instruments TA-Q50) was performed on a thermal analysis from 30 to 900 °C under oxygen atmosphere by heating the sample at a heating rate of 5 °C min^−1^. UV-vis absorption spectrum was measured on a JASCO V-660 spectrophotometer at room temperature. The adsorption and desorption measurements were done with a BELSORP-max instrument with nitrogen. The BET surface areas were calculated using the adsorption data in a relative pressure range of *p*/*p*_0_ = 0.05–0.10. The pore size distributions were calculated with the BJH method. Prior to each sorption measurement, the sample was degassed at 300 °C for 2 h under a vacuum to remove all the impurities completely.

### Data availability

All data generated or analysed during this study are included in this published article and its Supplementary Information file.

## Electronic supplementary material

Supplementary Information
