# Supplementary material for: Au nanoparticle@hollow mesoporous carbon with FeCo/graphitic shell nanoparticls as a magnetically recyclable yolk–shell nanocatalyst for catalytic reduction of nitroaromatics
Source: Sci Rep. 2018 May 10;8:7469. doi: 10.1038/s41598-018-25795-w (PMC5945776; doi:10.1038/s41598-018-25795-w)
Supplement: Supplementary file 1 — Supplementary Information [file 41598_2018_25795_MOESM1_ESM.pdf]

Supplementary Information for

Au nanoparticle@hollow mesoporous carbon with FeCo/graphitic shell nanoparticls as a magnetically recyclable yolk-shell nanocatalyst for catalytic reduction of nitroaromatics

Yonghoon Hong, In Ae Choi & Won Seok Seo

Department of Chemistry, Sogang University, Seoul, 04107, Republic of Korea. Correspondence and requests for materials should be addressed to W.S.S. (email: wsseo@sogang.ac.kr)

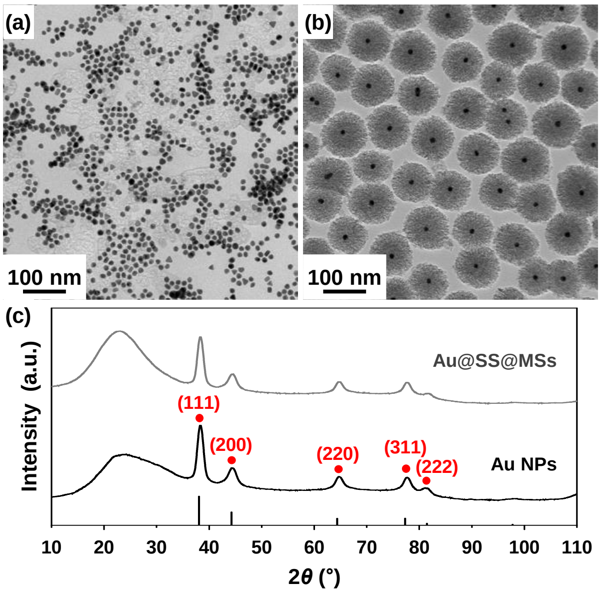

Supplementary Figure S1. (a, b) TEM images and (c) XRD patterns of Au NPs (a) and Au@SS@MSs (b).

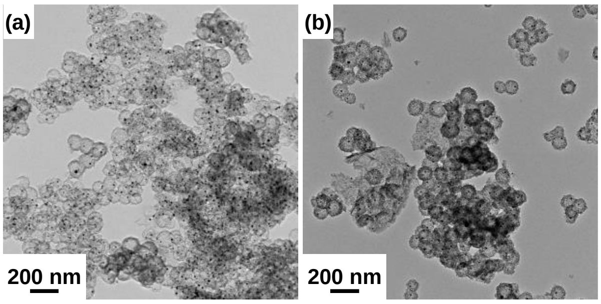

Supplementary Figure S2. TEM images of Au@hmCs prepared at the reaction temperature of 800 °C and the ethylene flow time of (a) 15 min and (b) 25 min, respectively.

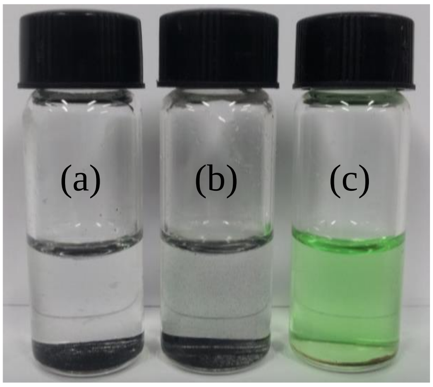

Supplementary Figure S3. Photographs of 35% HCl solutions of Au@hmC-FeCo/GCs (a) after being stored over a monitoring period of six months and after being heated at (b) 300 °C and (c) 550 °C in air for 1 h, respectively.

Supplementary Table S1. Physicochemical properties of Au@hmCs and Au@hmC-FeCo/GCs.

Sample	BET surface area ($m^2\ g^{-1}$)	Pore volume ($cm^3\ g^{-1}$)	Pore size (nm)
Au@hmCs	418.7	0.93	3.7
Au@hmC-FeCo/GCs	276.2	0.74	3.5

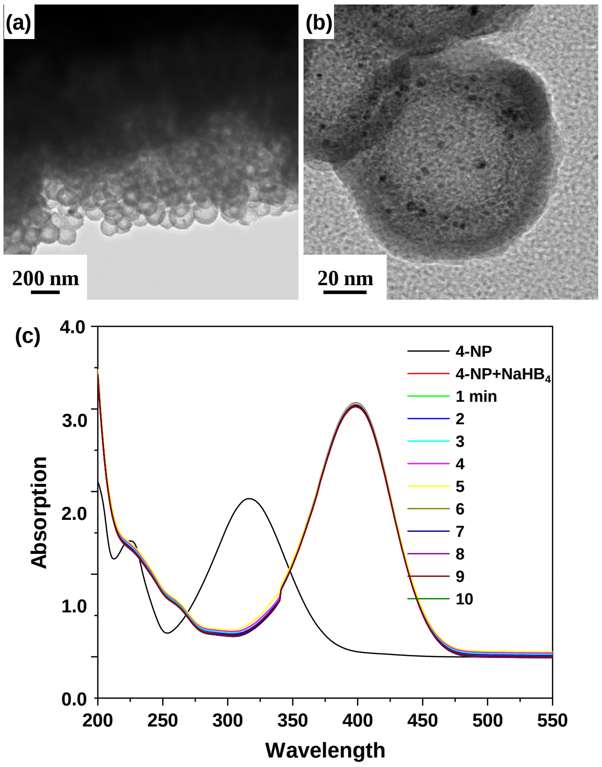

Supplementary Figure S4. (a, b) TEM images of hmC-FeCo/GCs. (c) UV-vis spectra of 4-nitrophenol (4-NP) before and after the addition of $NaBH_4$ in the presence of hmC-FeCo/GCs.

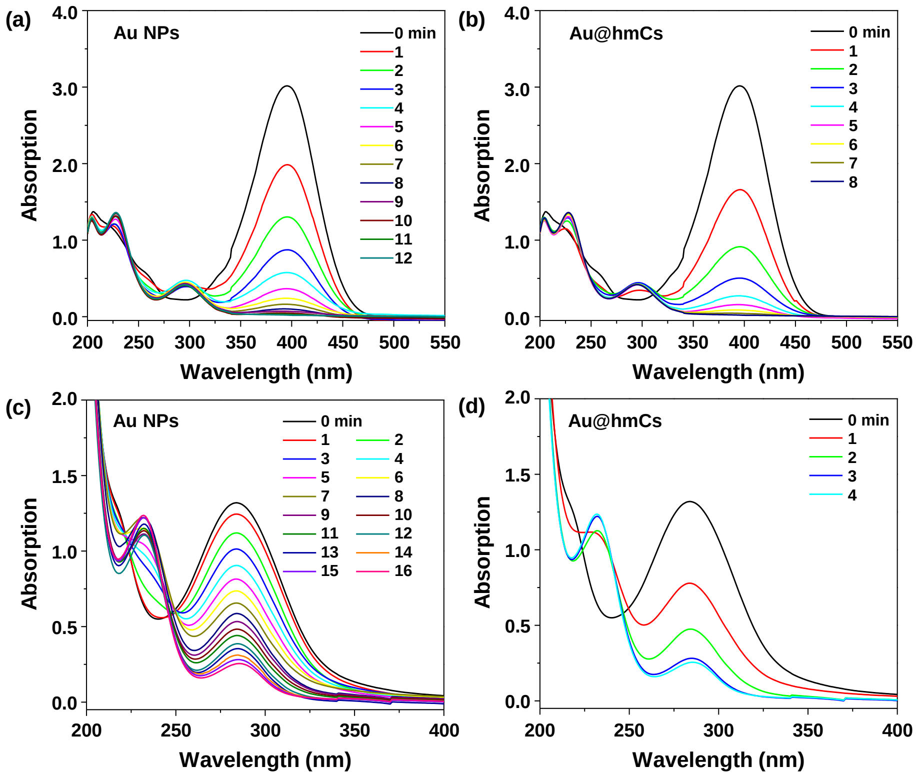

Supplementary Figure S5. Time-dependent UV-vis spectral changes of the reaction mixture catalysed by (a, c) Au NPs and (b, d) Au@hmCs for 4-nitrophenol (a, b) and 4-nitrotoluene (c, d).

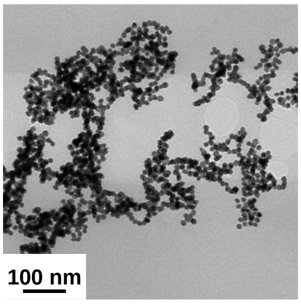

Supplementary Figure S6. TEM image of Au NPs after the first 4-nitrophenol reduction reaction.

Supplementary Table S2. Comparison of catalytic activities of various Au-based nanocatalysts for the reduction of 4-nitroaromatics.

Catalyst	Nitroaromatics	Nitroaromatic concentration (mM)	Mole ratio of Au:nitroaromatic:$NaBH_4$	Size of Au (nm)	K (min^{-1})	Reference
Au/graphene	4-NP	0.093	1:4:1642	14.6	0.19	1
Fe_3O_4@SiO_2-Au@mSiO_2	4-NP	0.25	1:1.5:597	12	0.35	2
Fe_3O_4@SiO_2-LBL-Au	4-NP	0.167	1:202:80645	4	0.34	3
Fe_3O_4@SiO_2@P(4VP-DVB)@Au	4-NP	0.45	1:532:212800	5	0.35	4
PNIPAM/Au@SiO_2	4-NP	0.0375	1:1.5:150	5	0.6	5
Polydopamine-Au	4-NP	50	1:6038:452898	2.8	0.057	6
SiO_2@Fe_3O_4/C@Au	4-NP	0.5	1:58:23200	2	0.97	7
Au/Co_3O_4	4-NP	0.1	1:105:15395	3.1	0.3	8
Graphene oxide@NH_2-Au	4-NP	0.067	1:22:1753	14	2.14	9
Au@Fe_3O_4 yolk-shell	4-NP	0.13	1:50:2500	2.5	0.94	10
Au@Fe_3O_4 yolk-shell	4-NT	0.13	1:50:2500	2.5	1.75	10
Au@hmC	4-NP	0.164	1:202:20202	13.3	0.60	This study
Au@hmC-FeCo/GC	4-NP	0.164	1:202:20202	13.3	0.55	This study
Au@hmC	4-NT	0.164	1:202:20202	13.3	0.79	This study
Au@hmC-FeCo/GC	4-NT	0.164	1:202:20202	13.3	0.74	This study

References

[1] Li, J., Liu, C. Y. & Liu, Y. Au/Graphene Hydrogel: Synthesis, Characterization and Its Use for Catalytic Reduction of 4-Nitrophenol. *J. Mater. Chem.* **22**, 8426−8430 (2012).

[2] Deng, Y. H. *et al.* Multifunctional Mesoporous Composite Microspheres with Well-Designed Nanostructure: A Highly Integrated Catalyst System. *J. Am. Chem. Soc.* **132**, 8466−8473 (2010).

[3] Zhu, Y. H. *et al.* Multifunctional Magnetic Composite Microspheres with in Situ Growth Au Nanoparticles: A Highly Efficient Catalyst System. *J. Phys. Chem. C* **115**, 1614−1619 (2011).

[4] Guo, W. C. *et al.* Fabrication of Hierarchical Fe_3O_4@SiO_2@p(4VP-DVB)@Au Nanostructures and Their Enhanced Catalytic Properties. *Chem. Asian J.* **10**, 701−708 (2015).

[5] Chen, Z. *et al.* Temperature-Responsive Smart Nanoreactors: Poly (n-Isopropylacrylamide)-Coated Au@mesoporous-SiO_2 Hollow Nanospheres. *Langmuir* **28**, 13452−13458 (2012).

[6] Ni, Y. Z. *et al.* One-Pot Preparation of Pomegranate-Like Polydopamine Stabilized Small Gold Nanoparticles with Superior Stability for Recyclable Nanocatalysts. *RSC Adv.* **6**, 40698−40705 (2016).

[7] Zeng, T. *et al.* A Double-Shelled Yolk-Like Structure as an Ideal Magnetic Support of Tiny Gold Nanoparticles for Nitrophenol Reduction. *J. Mater. Chem. A* **1**, 11641−11647 (2013).

[8] Yang, Y. W. *et al.* Facile Synthesis of Cubical Co_3O_4 Supported Au

Nanocomposites with High Activity for the Reduction of 4-Nitrophenol to 4-Aminophenol. *RSC Adv.* **6**, 32430−32433 (2016).

[9] Ju, Y. Y. *et al.* One Pot In-Situ Growth of Gold Nanoparticles on Amine-Modified Graphene Oxide and Their High Catalytic Properties. *Appl. Surf. Sci.* **316**, 132−140 (2014).

[10] Lin, F. H. & Doog, R. A. Catalytic Nanoreactors of Au@Fe_3O_4 Yolk−Shell Nanostructures with Various Au Sizes for Efficient Nitroarene Reduction. *J. Phys. Chem. C* **121**, 7844−7853 (2017).
